# Assessment of microvascular rarefaction in human brain disorders using physiological magnetic resonance imaging

**DOI:** 10.1177/0271678X221076557

**Published:** 2022-01-26

**Authors:** Maud van Dinther, Paulien HM Voorter, Jacobus FA Jansen, Elizabeth AV Jones, Robert J van Oostenbrugge, Julie Staals, Walter H Backes

**Affiliations:** 1 Department of Neurology, Maastricht University Medical Center, The Netherlands; 2 CARIM – School for Cardiovascular Diseases, Maastricht University, The Netherlands; 3 Department of Radiology and Nuclear Medicine, Maastricht University Medical Center, The Netherlands; 4 MHeNs – School for Mental Health and Neuroscience, Maastricht University, The Netherlands; 5 Department of Cardiovascular Sciences, KU Leuven, Leuven, Belgium

**Keywords:** Alzheimer’s disease, cerebral small vessel disease, magnetic resonance imaging, microvascular density, microvascular rarefaction

## Abstract

Cerebral microvascular rarefaction, the reduction in number of functional or structural small blood vessels in the brain, is thought to play an important role in the early stages of microvascular related brain disorders. A better understanding of its underlying pathophysiological mechanisms, and methods to measure microvascular density in the human brain are needed to develop biomarkers for early diagnosis and to identify targets for disease modifying treatments. Therefore, we provide an overview of the assumed main pathophysiological processes underlying cerebral microvascular rarefaction and the evidence for rarefaction in several microvascular related brain disorders. A number of advanced physiological MRI techniques can be used to measure the pathological alterations associated with microvascular rarefaction. Although more research is needed to explore and validate these MRI techniques in microvascular rarefaction in brain disorders, they provide a set of promising future tools to assess various features relevant for rarefaction, such as cerebral blood flow and volume, vessel density and radius and blood-brain barrier leakage.

## Introduction

Normal brain function requires a very high energy supply, as is illustrated by the fact that the brain uses about 20% of total body energy even though it comprises only 2% of total body mass.^1^ The human cerebral microcirculation consists of approximately 600 kilometers of capillaries, and the number of endothelial cells in the brain is very similar to the number of neurons.^2^ Since the brain has little reserve capacity, a reduction in perfusion is deleterious. There is growing evidence that alterations at the level of the cerebral microcirculation play a key role in vascular brain diseases and neurodegeneration.^3,4^

**Figure 1.**
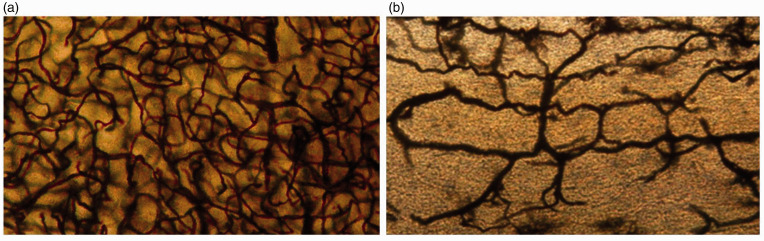
Electron-microscopic view on brain tissue obtained at autopsy. Capillaries from the CA1 area of the hippocampus, from a human who was neurologically intact (a). Capillaries from the CA1 area of the hippocampus, from a human who suffered from AD (b). The capillary density is substantially decreased in the AD patient (b) in comparison with the healthy control (a). Both images were taken at 1200x magnification. Source: reproduced with permission form publisher (2012).^52^

Importantly, small vessels such as arterioles and capillaries undergo a reduction in their number, a phenomenon called rarefaction (Figure 1).^5^ Microvessel density decreases with normal aging, but this process is accelerated in microvascular brain disorders.^6^ In pathology studies on post-mortem specimens, cerebral microvascular rarefaction has been demonstrated in animal models of ageing, Alzheimer’s disease (AD), obesity, hypertension and diabetes mellitus, and the same has been found in aging humans, AD patients, and humans with white matter hyperintensities (WMH).^6^

Microvascular rarefaction may reveal itself as a functional or structural phenomenon. Functional rarefaction reflects a reversible reduction in perfused microvessels, whereas structural rarefaction reflects an actual anatomical loss of microvessels.^7^ A reduction in perfusion likely precedes structural rarefaction.^8^ Reduced perfusion may eventually lead to neural dysfunction and microstructural tissue damage,^9^ which contribute to neurodegeneration and macrostructural lesions visible on magnetic resonance imaging (MRI).

Investigating microvascular density directly in the human brain is challenging, since pathological examination is only possible post-mortem, and the spatial resolution of the existing medical imaging machines is insufficient for direct visualization of cerebral capillaries as these are only about 10 µm in diameter. Nonetheless, since structural rarefaction is preceded or paralleled by functional rarefaction and microvascular dysfunction, this opens up avenues for measuring rarefaction by assessing microvascular perfusion and function.

In this narrative review, we provide an overview of the literature on microvascular rarefaction in the human brain. A better understanding of the relevance of microvascular rarefaction, its underlying mechanisms, and methods to measure microvascular density in the brain, is needed to develop markers for early diagnosis and to identify targets for disease modifying treatments. We will first discuss pathophysiologic mechanisms underlying cerebral microvascular rarefaction, and evidence of microvascular rarefaction in several brain disorders. Thereafter, relevant principles of advanced MRI techniques that can be used to study rarefaction in the human brain including their limitations are explained. We have limited the scope of this review to MRI approaches that probe physiologic properties in human vascular disease and aging, since MRI is the most promising and versatile imaging technique to measure rarefaction, as it can be repeated over time to monitor changes, and can provide a variety of functional measures on the microcirculation.

## Pathophysiology

**Figure 2.**
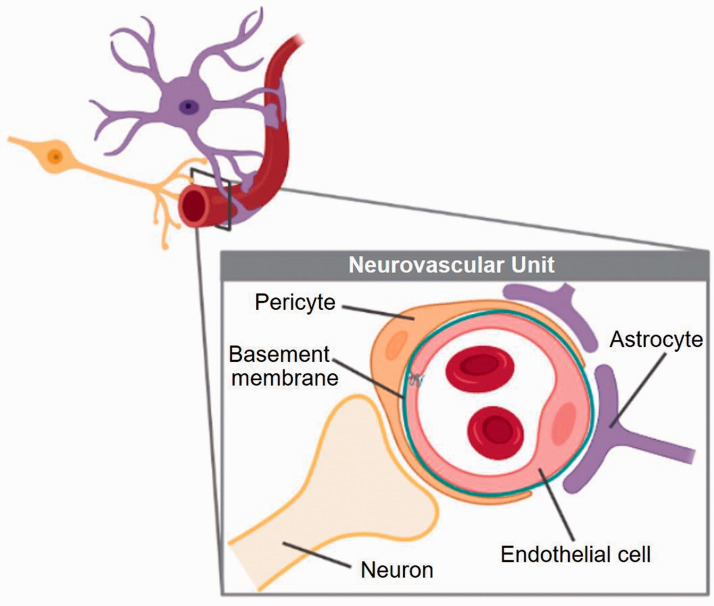
The neurovascular unit regulates the close relationship between metabolic demand and neuronal activity in the brain, and is composed of the vascular endothelium, pericytes, astrocytes, neurons, and extracellular matrix around the vessels.^10^ The components are intimately and reciprocally linked to each other, establishing an anatomical and functional unity. The blood-brain barrier, which is formed by the endothelial cells connected by tight junctions, pericytes and astrocytes, protects the neuronal microenvironment by separating harmful components of the blood circulation from neurons, and by maintaining the chemical composition of the neuronal environment. Neurons detect variations in oxygen and nutrients supply, and subsequently communicate with the vessels through astrocytes, thereby influencing the vascular tone. Anatomically, neurovascular communication takes place through the astrocyte endfoot, which is a highly specialized astrocyte extension that is in contact with the surface of pericytes. Functions of pericytes include stabilizing the vascular wall and maintaining vascular quiescence and vascular integrity by bidirectional signaling with endothelial cells, deposition of the basement membrane, providing capillary contraction, and bridging endothelial gaps. Endothelial cells enclose the vascular lumen, and produce trophic and vasoactive factors important for vascular health and tone. *Image was created by use of biorender*.

**Figure 3.**
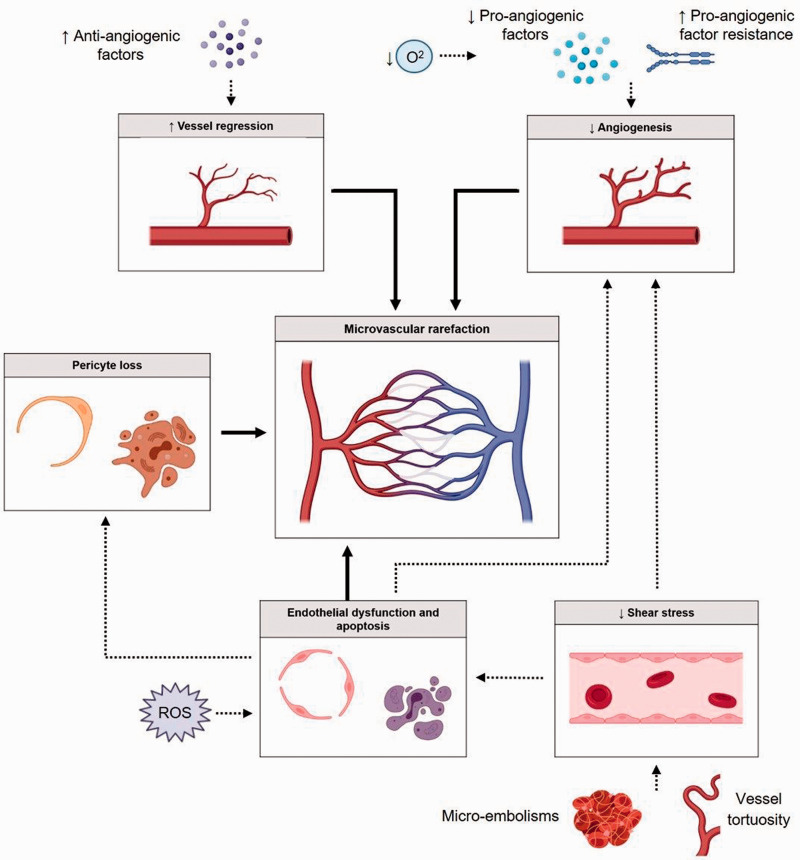
Schematic overview of the pathophysiological processes leading to microvascular rarefaction. Increased levels of anti-angiogenic factors induce vessel regression, directly resulting in microvascular rarefaction. An insufficient increase in pro-angiogenic factors, as well as a resistance towards pro-angiogenic factors, prevent adequate angiogenesis during hypoxia. Oxidative stress results in endothelial dysfunction and apoptosis, both directly leading to microvascular rarefaction. Furthermore, endothelial dysfunction and apoptosis also indirectly contribute to rarefaction due to the disturbance in pericyte-endothelial cross-talk leading to pericyte loss, and via its suppressive effect on angiogenesis. Pericyte loss directly leads to rarefaction by leaving the vascular wall unprotected and vulnerable, by the transition to myofibroblasts which promote the loss of capillaries, and by the loss of the reserve capacity to replenish vascular cells lost during aging. Micro-embolisms and increased vessel tortuosity result in decreased shear stress, indirectly leading to microvascular rarefaction by inducing endothelial dysfunction and apoptosis, as well as impaired angiogenesis. *Image was created by use of biorender*.

Though different processes likely involved in the reduction of microvessels have been identified, the exact pathophysiology underlying microvascular rarefaction in the brain is as yet unknown. Most of these processes involve changes in the cells composing the neurovascular unit,^10^ which is a constellation of microstructural elements that regulates the close relationship between metabolic demand, blood supply and neuronal activity in the brain (Figure 2). We discuss four pathological processes which most likely play an important role in microvascular rarefaction. A schematic overview of the pathophysiological processes resulting in rarefaction is presented in Figure 3. Most knowledge about the pathophysiological processes has been acquired through histologic analysis on specimens from animal studies, and to a lesser extent through analysis of human pathological post-mortem samples.

### Impaired angiogenesis and active capillary regression

Pro-angiogenic factors, such as vascular endothelial growth factor (VEGF) and insulin like growth factor-1 (IGF-1), are essential for endothelial cell survival and proper microvascular function, and are known to be dysregulated in microvascular rarefaction.^11^ Vascular density is directly proportional to levels of these proteins. Indeed, increased expression of VEGF or IGF-1 in rodents is accompanied by an increase in cerebrovascular capillary density,^12–15^ whereas low levels of these pro-angiogenic substances are associated with microvascular rarefaction.^13,16^ However, cerebral microvascular endothelial cells derived from aged rodents exhibit an impaired angiogenic response to exogenous administration of VEGF.^17^ This suggests that endothelial cells becoming resistant to inducers of angiogenesis, rather than a decline in serum levels of pro-angiogenic factors, is critical in the pathophysiology of rarefaction.^18^

**Figure 4.**
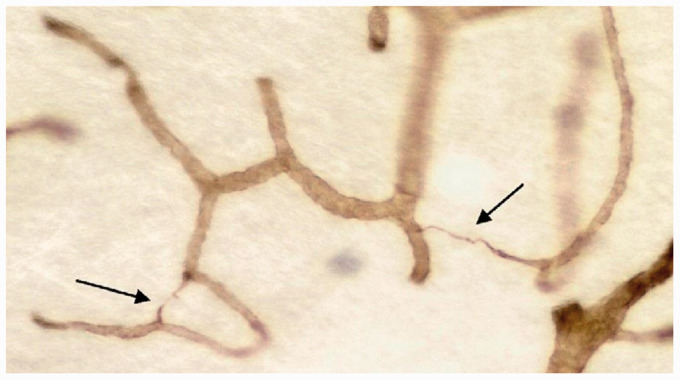
String vessels (arrows) which represent basement membranes without endothelial cells in a thick celloidin section of the cerebral white matter, stained with antibody to collagen IV. Source: reproduced with permission form publisher (2009).^31^

In the adult human brain, however, the vasculature is relatively quiescent and angiogenesis only occurs in response to hypoxia.^19^ This implies defects in angiogenesis cannot account for the initiation of capillary rarefaction, though they can account for failed repair response after microvascular rarefaction has occurred and a state of hypoxia is present. Active capillary regression is now increasingly being recognized as important in microvascular rarefaction though its molecular mechanisms remain poorly understood. Actively regressing vessels can be identified by string vessels, which are basement membranes without endothelial cells (Figure 4).^20^ Although regression is a normal part of development, improper activation of regression signals can lead to microvascular rarefaction, and this process could account for the commencement of it in the absence of hypoxia.

Active regression is not simply the result of a lack of pro-angiogenic factors. Indeed, merely reducing VEGF is not sufficient to initiate or induce capillary regression, and even in the presence of relatively high levels of VEGF, vessel regression occurs.^21^ By contrast, negative angiogenic regulators may be the key determinant in initiating and regulating capillary regression.^21,22^ For example, a study using transgenic mice that overproduce a constitutively active form of transforming growth factor-β1 (TGF-β1) showed an increased number of string vessels in the cortex and hippocampus in comparison with wild type mice.^23^ Of note, TGF-β1 is shown to be increased in the cerebrospinal fluid and blood of AD and vascular cognitive impairment (VCI) patients.^24,25^ Furthermore, post-mortem studies that compared the brains of AD patients with elderly controls have shown string vessels to be increased in AD.^26–30^ On the contrary, string vessels were found to be decreased in cerebral white matter in subjects with WMH.^31^ However, since it is known that string vessels gradually disappear with time, and the observed decrease in string vessels was greater than the decrease in capillaries, it was hypothesized that an early loss of capillaries in WMH is followed by the disappearance of string vessels, so that by the time the subject dies there are few string vessels left.^31^

### Endothelial dysfunction and apoptosis

Cumulative exposure to cardiovascular risk factors leads to oxidative stress by reactive oxygen species (ROS), causing endothelial dysfunction and apoptosis.^32^ Endothelial dysfunction has been shown to correlate with functional rarefaction,^33^ and apoptotic endothelial cell death is involved in structural capillary rarefaction.^34^ Endothelial injury causes a decreased bioavailability of nitric oxide (NO), resulting in a reduced arteriolar vasodilator capacity, leading to decreased blood flow through the capillaries, i.e. functional rarefaction.^35^ Decreased NO production also promotes endothelial apoptosis,^36^ and has been causally linked to an impaired angiogenic capacity of endothelial cells,^37^ thereby directly contributing to structural rarefaction as well. Apoptosis of a relatively small proportion of endothelial cells may be sufficient to mediate significant microvessel rarefaction.^38^ The importance of oxidative stress in the origin of endothelial injury in the brain is supported by studies showing that attenuation of mitochondrial oxidative stress using different inhibitors of ROS production increases cerebral capillary density, restores angiogenic potential in aged rodents,^39,40^ and restores endothelium-mediated vasodilation in the brain of aged mice.^41,42^

### Pericyte loss

One of the functions of pericytes is to control capillary tone. Whereas enhanced activity of pericytes causes capillary contraction and entrapment of erythrocytes,^5^ the loss of pericytes results in dilated capillaries, leading to uneven and disrupted flow patterns within the network, causing disruptions in capillary transit time.^43^ Thus, pericytes may play an important role in the functional stage of microvascular rarefaction. Furthermore, when a disturbance in the pericyte-endothelial cross-talk appears, pericytes detach from the vascular wall and migrate into the interstitial space, leaving the vascular wall unprotected and vulnerable.^44^ In the interstitial space, pericytes undergo a functional transition to myofibroblasts (scar-forming cells) ^45^ or undergo apoptosis.^46^ Preliminary studies have shown that myofibroblasts may promote vascular leakage and the death of capillaries.^47,48^ Hence, pericyte detachment is deleterious to capillaries in multiple ways and the loss of pericytes results in structural rarefaction. Pathways involved in pericyte-endothelial cell disassembly have been reviewed in detail before and will not be discussed here further.^47^ Lastly, pericytes might serve as a local stem cell population that replenish differentiated interstitial and vascular cells lost during aging.^49^ The loss of this reparative capacity also promotes capillary rarefaction. Indeed, in mice models, a reduced pericyte coverage and pericyte number in the brain have repeatedly been correlated with reduced microvascular density.^18,50,51^ A postmortem study showed a reduced microvessel density in combination with marked degeneration of pericytes in the hippocampus and cortex in AD patients in comparison with controls.^52^

### Loss of shear stress

Stagnation of blood flow leading to a reduction of shear stress can be caused by tortuosity of microvessels and vascular blockage by spontaneous microemboli. Tortuosity of microvessels occurs with aging ^53^ and is associated with cardiovascular risk factors such as hypertension and diabetes.^54,55^ Microemboli are associated with both AD and vascular dementia.^56^ Decreased shear stress leads to compromised endothelial NO synthase (eNOS) function in endothelial cells resulting in lower levels of NO, which initially leads to prolonged periods of functional rarefaction by compromised vasodilation, and eventually results in structural rarefaction.^5^ Additionally, mechanical forces may play a significant role in pro-angiogenic and anti-angiogenic reprogramming. Cyclic strain triggers endothelial cell programs which result in sprouting angiogenesis and recruitment of smooth muscle cells.^57^ For example, it has been shown that a lack of blood flow results in loss of availability of VEGF.^58^ Flow dependent shear forces also provide the survival signal for endothelial cells and a decrease in shear stress in capillaries facilitates endothelial apoptosis.^59^ As such, it has been shown that shear stress generated by a flow chamber attenuates brain microvascular endothelial cell apoptosis in rats.^36^ Hence, a loss of shear stress may present a mechanism linking functional and structural rarefaction.

### Linking the different pathophysiological mechanisms

To provide a structured overview of the pathophysiological pathways involved in brain microvascular rarefaction, we presented the four main pathophysiological processes separately. However, these processes are not self-containing, yet there is a continuous interplay between these processes. Pericytes, for example, are dependent on cross-talk with endothelial cells for proper functioning, so endothelial dysfunction or apoptosis will typically be accompanied by pericyte loss.^47^ Endothelial cells are also the key cell type involved in angiogenesis, hence endothelial malfunction or death will also result in impaired angiogenesis.^37^ A decrease in shear stress, on its turn, leads to impaired angiogenesis and endothelial dysfunction and apoptosis,^57,59^ rather than directly causing rarefaction. This interdependency leads to self-reinforcement, where dysfunction in one cell type sets of a chain of events causing further acceleration of the pathophysiological process. Although a complex interaction between these processes is probably present, most studies have mainly focused on a singular process. It is for this reason that the exact sequence and relative importance of events in the origin of microvascular rarefaction in the human brain remains elusive.

Furthermore, most evidence for these pathophysiological processes of microvascular rarefaction in the brain comes from animal studies, which can be regarded as low level evidence, since it is unclear how findings in animals relate to findings in humans. Although far more research has been conducted in impaired angiogenesis than in pericyte loss in animal models, pericyte loss and vessel regression have been confirmed in human post-mortem case-control studies.^26–30,52^ On the other hand, for impaired angiogenesis, endothelial dysfunction and loss of shear stress, ‘intervention’ studies in animals have been performed to intervene in the pathophysiological process resulting in higher microvascular density,^12–15,36,39,40^ and these could be regarded as a high level of evidence for the role of these processes.

## Evidence for microvascular rarefaction in microvascular brain disorders

### Cerebral small vessel disease

cSVD is an umbrella term that covers all pathologies of the cerebral small vessels. It causes hemorrhagic strokes, ischemic lacunar strokes, VCI and dementia.^60^ One of the typical structural brain lesions that can be seen on MRI are WMH.^61^ The precise pathways in microvascular dysfunction leading to cSVD remain unclear, but there is growing evidence that microvascular rarefaction plays a contributing role. Postmortem studies showed that subjects with WMH had a decreased cerebral microvascular density in comparison with subjects without WMH.^62,63^ In a mouse model of cerebral autosomal dominant arteriopathy with subcortical infarcts and leukoencephalopathy (CADASIL), a mono-genetic type of cSVD, a substantial reduction in white matter capillary density was shown, and this rarefaction was shown to precede the occurrence of WMH and to be progressive.^64^ A decline in cerebral microvascular density has also been correlated with diminished cognitive function in mice.^18^

Additionally, microvascular rarefaction in the brain has been shown in rodents with cardiovascular risk factors, such as hypertension, diabetes, and obesity.^65–70^ Since these comorbidities are strongly linked to the development of age- and vascular risk factor associated cSVD and have been shown to cause cerebral microvascular rarefaction, this also supports a role of rarefaction in the development of cSVD.

### Alzheimer’s disease

AD, a neurodegenerative brain disease, is the most common cause of dementia in the elderly. Although deposition of amyloid β (Aβ) and hyperphosphorylated misfolded tau have long been the focus in explaining the molecular cause of AD, accumulating evidence now suggests that the early stage of AD might be primarily a microvascular disorder.^4^ Aβ depositions are present in the neurovascular unit and their presence is associated with microvascular impairment, which contributes to the development of early-stage pre-plaque cognitive dysfunction as well as subsequent progression of the disease.^4,71^ Furthermore, increased deposition of Aβ results in degeneration and disappearance of capillaries and small vessels, i.e. microvascular rarefaction.^72,73^ Subsequently, capillary loss leads to an impaired clearance of Aβ, which further promotes vascular damage.^74^ Aβ accumulations on capillaries may contribute to the reduced brain capillary density via endothelial apoptosis due to its toxic effects,^75,76^ and via its intrinsic anti-angiogenic activity^77^ and capacity to bind other pro-angiogenic factors such as VEGF.^78^ Several studies have revealed a decreased microvascular density and an increased number of string vessels in the brain of AD patients.^26,62,79–85^

### Ageing

Ageing may not be considered a pathology, but its processes show considerable overlap with cSVD and neurodegeneration. Numerous studies have shown a decreased cerebral microvascular density in the brain of healthy aging humans.^26,62,79–81,86,87^ Although microvascular density is lower in younger AD and cSVD cases in comparison with age matched controls, microvascular density in these patient groups and controls converges by the ninth decade of life.^6,62^ Since microvascular density also decreases with age in absence of disease, it can be challenging to distinguish the effect of ageing from disease specific processes in cSVD and neurodegeneration.

### Cognitive impairment following whole brain radiation therapy

Whole brain radiation therapy leads to cognitive impairment in 40-50% of brain tumor survivors.^88^ Although the etiology of cognitive deficits post whole brain radiation therapy remains unclear, microvascular rarefaction appears to be an important contributor. Indeed, whole brain radiation therapy induced profound microvascular rarefaction in rats and mice,^89,90^ and a reduced capillary density in the hippocampus of these mice is linked to deficits in learning and memory.^91^ It has been shown that brain irradiation induces dose-dependent endothelial apoptosis, loss of pericytes,^92^ and impaired angiogenesis,^93^ all promoting rarefaction. Therapeutic strategies, such as chronic systemic hypoxia, restore capillary density and this results in the rescue of cognitive function.^89^

## Microvascular rarefaction: cause or consequence?

During ageing and disease progression, both brain capillaries and neural tissue are lost. Whereas loss of neural tissue could result in a decreased need for oxygen and glucose and subsequently might lead to capillary loss, several studies have shown an increased oxygen extraction from the blood in WMH, suggesting capillary loss occurs first.^94–96^ In conjunction with this, studies have not only revealed a decreased cerebral blood flow (CBF) in WMH, but also in undamaged brain regions.^64,97^ Likewise, a CBF reduction was already detectable before any brain lesions appeared.^64^ These findings support a causal role of hypoperfusion rather than a secondary reduction of perfusion due to a reduced metabolic demand of damaged tissue in the brain.

## MRI methods to measure microvascular rarefaction

This section explains and discusses advanced MRI techniques, which can provide measures that reflect certain aspects of the pathophysiological pathways associated with microvascular rarefaction in human. Individual capillaries cannot be visualized morphologically and the various pathophysiological processes cannot be visualized directly. Instead, MRI can give a proxy by measuring their functional characteristics such as microvascular volume and flow, wall permeability, cerebrovascular reserve capacity, and also sense various sizes of small blood vessels. These functional MRI measures do not reflect properties of individual microvessels, but the combined effect of a large number of microvessels in a tissue region with a size of one or a few cubic millimeters. A summary of the discussed MRI techniques is provided in Table 1. Moreover, in Table 2 we present an overview of how these microvascular MRI measures are expected to relate to the four main pathophysiological processes discussed above.

**Table 1.** Summary of MRI techniques.

MRI technique	Principles	Primary output measures	Additional output measures	Contrast agent administration	Measurement of blood supply
DSC-MRI	Dynamically measures magnetic susceptibility changes during the first passage of contrast agent	Cerebral blood flow, cerebral blood volume, mean transit time	Capillary transit time heterogeneity	Yes	Yes, arterial input function
Vessel size imaging	Provides the average radius of the small blood vessels within a voxel by measuring the ratio between ΔR2 and ΔR2*	Mean vessel diameter, vessel size index (or vessel radius), mean vessel density	–	Yes	No
DCE-MRI	Measures the leakage rate of contrast agent particles from the blood into the tissue by using a dynamic T1-weighted measurement	Blood-brain barrier leakage rate	Blood volume fraction and flow	Yes	Yes, arterial or alternatively venous input function
ASL	Measures cerebral blood flow by inverting blood water spins and measuring this labeled blood when it flows through tissue after a delay time	Cerebral blood flow	Water exchange rate over blood-brain barrier, cerebral blood volume, mean transit time, arterial transit time	No	No, but blood in supplying artery is magnetically labeled
IVIM	Simultaneously measures parenchymal diffusion and microvascular pseudo-diffusion, which is considered to represent the blood flow through a random network of capillaries	Parenchymal diffusion, microvascular pseudodiffusion, microvascular perfusion fraction	–	No	No

MRI: magnetic resonance imaging; DSC: dynamic susceptibility contrast-enhanced; DCE: dynamic contrast-enhanced; ASL: arterial spin labeling; IVIM: intravoxol incoherent motion imaging; R2: relaxation rate.

**Table 2.** Expected contributions of pathophysiological effects of rarefaction to physiological MRI measurements.

Pathophysiology	Effect	Expected effect detectable by MRI	MRI technique
Impaired angiogenesis and capillary regression	Reduced vessel surface area	Decreased S, thus decreased K^trans^	DCE MRI
	Reduced blood flow and volume	Decreased CBF and CBV	DSC MRI, ASL and IVIM
	Loss of capillaries	Decreased mean vessel density	VSI
Endothelial dysfunctionEndothelial apoptosis	Decreased NO production and reduced vasodilation	Decreased CBF and CBV Decreased vessel size index and vessel size radius	DSC MRI, ASL and IVIMVSI
	BBB disruptionLoss of capillaries	Increased P, thus increased K^trans^Decreased mean vessel density	DCE MRIVSI
Enhanced pericyte contractionPericyte loss	Reduced blood flow and volumeVasoconstriction	Decreased CBF and CBVDecreased vessel size index and vessel size radius	DSC MRI, ASL and IVIMVSI
	BBB disruptionDilated capillaries causing uneven and disrupted flow patterns within the networkLoss of capillaries	Increased P, and thus increased K^trans^Increased CTTHIncreased vessel size index and vessel size radiusDecreased mean vessel density	DCE MRIDSC MRIVSIVSI
Loss of shear stress	Decreased NO production and reduced vasodilation	Decreased CBF and CBV Decreased vessel size index and vessel size radius	DSC MRI, ASL and IVIMVSI
	Impaired angiogenesis resulting in a reduced vessel surface area and reduced blood flow and volume	Decreased S, thus decreased K^trans^Decreased CBF and CBVDecreased mean vessel density	DCE MRIDSC MRI, ASL and IVIMVSI
	Endothelial apoptosis leading to BBB disruption and capillary loss	Increased P, thus increased K^trans^Decreased mean vessel density	DCE MRIVSI

BBB: blood-brain barrier; NO; nitric oxide; S: vessel surface area; P: permeability; CBF: cerebral blood flow; CBV; cerebral blood volume; CTTH: capillary transit time heterogeneity; K^trans^: blood-brain barrier leakage rate; MRI: magnetic resonance imaging; DCE: dynamic contrast-enhanced; DSC: dynamic susceptibility contrast-enhanced; ASL: arterial spin labeling; IVIM: intravoxol incoherent motion imaging; VSI: vessel size imaging.

### Dynamic susceptibility contrast-enhanced MRI

Dynamic susceptibility contrast-enhanced (DSC) MRI is a technique that measures hemodynamic characteristics, such as cerebral blood flow and volume. In the DSC MRI method, multiple spin echo (SE: T2-weighted) or gradient echo (GE: T2*-weighted) images are acquired with high temporal resolution during an intravenous bolus injection of a paramagnetic contrast agent (e.g. gadolinium). This dynamic measurement typically only captures the first passes of the contrast agent through the brain. The contrast agent particles change the local homogeneity of the magnetic field (cause T2 and T2* shortening) inside and in the vicinity of the blood vessels, thereby lowering the signal intensity. The amount of this signal attenuation is related to the contrast agent concentration, which enables converting the measured signal intensity curves to concentration curves over time. Subsequently, the quantification of CBF, cerebral blood volume (CBV) and mean transit time (MTT) of the contrast agent through the brain tissue can be obtained by applying a computational hemodynamic model to the tissue and arterial concentration curves, which also corrects for the blood supply. A more advanced DSC measure worth mentioning is the capillary transit time heterogeneity (CTTH), which reflects whether the capillary network is perfused homogenously.^43^

DSC-MRI is widely used in clinical research, since it provides a strong signal effect between the pre- and post-contrast signal and has a short acquisition time (2-3 min).^98^ Though DSC-MRI has been well established in clinical research, the process to obtain quantitative measures is complex and a thorough understanding of its acquisition parameters, image processing and limitations is needed to ensure accurate results. Most of these aspects concerning DSC-MRI have previously been reviewed.^99^

**Figure 5.**
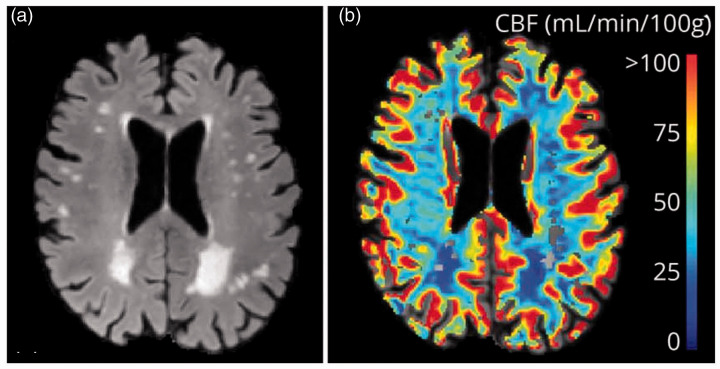
Fluid Attenuated Inversion Recovery of cSVD patient (A) and the corresponding CBF map measured by DSC MRI (B). The CBF is higher in the cortical grey matter and lower in WMH compared to normal appearing white matter. CBF: cerebral blood flow; cSVD: cerebral small vessel disease; DSC MRI: dynamic susceptibility contrast-enhanced magnetic resonance imaging; WMH: white matter hyperintesities.Source: reproduced with permission form publisher (2019).^106^

GE is different from SE sequences with respect to their sensitivity for vessel size. GE shows a stronger signal drop, since the GE signal is effected by the contrast agent in blood vessels of all sizes, whereas the SE signal is most sensitive to magnetic field inhomogeneities in and around the small blood vessels (radius <10 μm).^98,100^ Accordingly, SE is found to be more representative when measuring microvascular perfusion characteristics compared to GE,^101^ and thus is the favorable technique to detect alterations in capillary perfusion as a proxy for microvascular rarefaction. For example, the relative CTTH measured by SE was found to correlate with WMH load and cognitive impairment in an AD cohort, while these same correlations could not be shown when CTTH was measured by GE.^102^ However, due to the stronger signal modifying effect GE has previously been more frequently performed than SE. Using GE, hypoperfusion has been demonstrated in AD patients compared to controls.^103,104^ In contrast, however, few studies found hyperperfusion in prodromal AD (at the stage of mild cognitive impairment), which was hypothesized to be a compensatory mechanism.^103,104^ Furthermore, a strong negative correlation was found between brain perfusion measured with GE and cSVD-burden score.^105^ An example of a CBF map measured with DSC MRI in cSVD is shown in Figure 5.^106^

### Vessel size imaging

Vessel size imaging is an MRI technique that relies on measuring both a SE (for relaxation rate R2 = 1/T2) and GE (for R2* = 1/T2*) before and after contrast agent administration. Since SE is more sensitive for small vessels than GE, the ratio of changes in relaxation rates ΔR2 and ΔR2* is dependent on the density and the average radius of the small blood vessels, which can be calculated within a voxel by this technique.^107^ Two measurement approaches can be distinguished in vessel size imaging: steady state imaging and dynamic imaging.

The steady state approach acquires only two R2* and R2 measurements (one pre- and one post-contrast). During the post-contrast measurement, the contrast agent concentration in the blood needs to be stable, which is the reason that ultra-small paramagnetic iron oxide (USPIO) is a more suitable contrast agent for the steady state approach than gadolinium-based contrast agents (USPIOs have a slower clearance). The dynamic approach acquires high temporal SE and GE images before, during and after the contrast agent concentration peak. Hereafter, the pre- and post-contrast relaxation rates are calculated to obtain ΔR2* and ΔR2 curves over time. The dynamic approach is preferred in human research, since gadolinium based contrast agents have considerably less health risks than USPIOs and the acquisition period can be kept shorter.^100^

The measures that can be determined using vessel size imaging are the mean vessel density (Q), the mean vessel diameter (mVD) and the vessel size index (VSI) or vessel size radius. Q and mVD are related to the vessel architecture, however, they are also dependent on the acquisition parameters and on the amount of water diffusion in the brain, since the amount of parenchymal water diffusion is related to the amount of signal attenuation of the spin echo. The VSI, expressed in μm, is a diffusion-corrected measure, hence an additional MRI acquisition should be performed to determine the tissue diffusion coefficient. Recommendations for vessel size imaging parameters and analysis methods are explained elsewhere.^100^ Furthermore, we would like to point out that the fast dynamic (hybrid) GE-SE sequence can also produce CBV and CBF maps, assuming that the temporal resolution remains sufficient.^100^ Therefore, this technique can easily replace DSC-MRI.

Up to this point, human vessel size imaging research was mainly performed in patients with brain tumors, where two studies have demonstrated that the VSI of tumor tissue highly correlated with the true vessel radius, obtained by histopathology.^108,109^ Vessel size imaging has also recently been performed in patients with subcortical vascular dementia.^110^ In the white matter of these patients, a higher mVD and VSI was found compared to healthy controls, probably due to dilatation of small vessels and/or a relatively fast decreasing number of small vessels in dementia. Taken together, vessel size imaging seems to be a promising technique to reveal information about the microvascular architecture in-vivo, although it has not yet been investigated extensively in diseases associated with rarefaction.

### Dynamic contrast-enhanced MRI

The integrity of the cerebral microvascular walls can be measured with dynamic contrast-enhanced (DCE) MRI. This technique acquires dynamic T1-weighted images before, during and after gadolinium-based contrast agent administration. The gadolinium molecules lower the longitudinal relaxation time (T1), and therefore enhance the T1-weighted signal.

**Figure 6.**
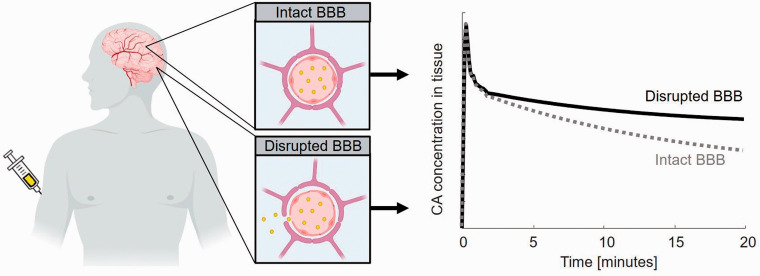
Principle of assessing the BBB leakage. The contrast agent remains intravascular when the BBB is intact, whereas the contrast agent leaks into the extracellular extravascular space when the BBB is disrupted. The contrast agent concentration in tissue decreases slower in case of impaired BBB due to the retention of contrast agent in tissue. *Image was created by use of biorender*. BBB: blood-brain barrier; CA: contrast agent.

When the blood-brain barrier (BBB) is disrupted, contrast agent particles are no longer fully restricted to the vascular compartment and can leak into the extravascular extracellular space of the brain tissue (Figure 6). Retention of contrast agent in brain tissue can be detected by converting signal enhancement curves to concentration curves over time in both tissue and blood. Quantification of the BBB leakage is then obtained by a pharmacokinetic model.^111^ Recommendations for DCE MRI acquisition parameters and analysis for subtle BBB impairment (e.g. in cSVD and AD), which differ from those used for high BBB leakage rates (e.g. in high-grade tumors and stroke lesions), were given elsewhere.^112^ For subtle BBB leakage, the simple pharmacokinetic graphical Patlak method is recommended, which neglects the reflux of contrast agent. The main output measure is the BBB leakage rate *K^trans^*, which is the product of the vessel surface area (S) and the permeability (P).^113^ An additional output measure of this model is the blood volume fraction (*v_b_*).

Although measuring BBB leakage with DCE MRI is promising, it has some limitations that impede the use of this technique in routine clinical practice. The main limitations are the significant image noise influencing the tissue concentration curves and consequently *K^trans^*, and the long scanning time (>15 minutes) to allow the contrast agent particles to extravasate from the blood into the tissue.^112^

DCE-MRI has previously revealed an increased BBB leakage rate in cSVD ^114,115^ and early AD.^116^ In cSVD, it has been demonstrated that a higher BBB leakage rate (measured with DCE-MRI) is correlated with decreased perfusion (measured with DSC-MRI),^106^ and with decreased cognitive performance.^115^ The BBB was also found to be increasingly impaired with (normal) aging.^117^

### Arterial spin labeling

Arterial spin labeling (ASL) can measure cerebral blood flow (CBF) by using inflowing water as an endogenous tracer. This technique inverts the magnetization of the blood water spins in the feeding arteries, thereby labeling the blood before it enters the brain. The brain tissue is imaged after a delay time, which allows the labeled spins to travel from the arterial compartment to the tissue capillary bed. For each labeled image, a corresponding control image is acquired. After image acquisition, the difference is calculated between the non-labeled and the labeled signal, which is proportional to the CBF.

**Figure 7.**
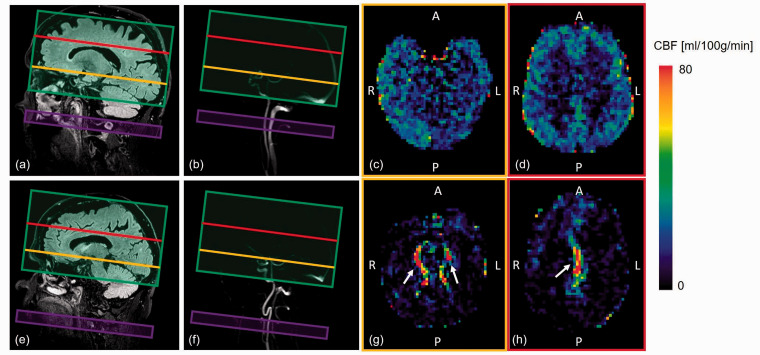
Examples of ASL planning and CBF maps for healthy subject (upper row) and a patient with vascular cognitive impairment (bottom row). The acquisition volume (green) captures the brain tissue (a) (e), and the labeling slice (purple) is placed orthogonally to the arteries (b) (f). The importance of visualizing the arteries for ASL planning is shown in (f), where the artery map is required to avoid the tortuous part of the artery in the labeling plane. The CBF maps for a healthy subject are shown in (c) and (d), where the labeled blood water spins have reached the brain tissue. The CBF maps for a patient with vascular cognitive impairment (lower arterial blood velocity) are shown in (g) and (h), where the labeled blood water spins are still located in the arteries (white arrow indicate the high perfusion signal from arteries). CBF: cerebral blood flow; A: anterior; P: posterior; R: right, L: left; ASL: arterial spin labeling.

Although several labeling and acquisition approaches exist, pseudo-continuous ASL (pCASL) is most commonly used in clinical research applications.^118^ In pCASL, a train of short RF pulses is applied to invert the spins flowing through a labeling plane that is positioned orthogonally to the artery (Figure 7). The length of this RF pulse train is called the labeling duration. Another important acquisition parameter in ASL imaging is the post-labeling delay (PLD), which is defined as the time between the end of the labeling period and the start of the tissue imaging. Since the labeled (i.e. inverted) blood water spins relax back to their equilibrium state, the labeled spins ‘lose’ their labels when more time has passed after the labelling period, and thus no perfusion signal is captured when the PLD is too long. However, when the PLD is too short, the flowing labeled spins have not yet entered the tissue of interest, so the optimal choice depends on the arterial transit time (ATT). For example, a longer ATT is expected for older adults (>70 years) and/or persons with tortuous vessels, which explains that the recommended PLD is longer for older than for younger adults.^119^ Thus, a correct PLD ensures that the flow signal is measured from the capillaries and not from the afferent and/or efferent blood vessels, but in practice the capillary flow cannot entirely be distinguished from the arteriolar flow.

ASL is an acknowledged MRI technique to measure CBF quantitatively in the grey matter. Consensus recommendations have been given for its clinical implementation.^120^ However, ASL has a lower contrast effect compared to DSC-MRI. The contrast effect of ASL is particularly low in white matter, since white matter has a longer ATT and lower perfusion compared to grey matter.^121^ Therefore, performing ASL at a magnetic field of 3 T or higher is strongly preferred, as this increases the contrast-to-noise ratio.^119^ Although ASL is ready for clinical use, new variants are still emerging to address the remaining challenges^122^ and to provide additional physiological measures, such as the rate of water exchange across the BBB.^123^

Since ASL is non-invasive and can easily be repeated, this technique is well suited to measure another relevant biomarker reflecting the microvascular condition: cerebrovascular reserve (CVR). To determine CVR, CBF is measured in both resting state and maximum flow state (i.e. during a vasodilator challenge). This maximum flow state can be created physiologically (breath hold or CO2 inhalation) or pharmaceutically (acetazolamide administration). The CVR describes to what extent the CBF is able to increase during the vasodilator challenge compared to baseline. In patients with structural rarefaction, most residual capillaries are already perfused and dilated at rest, so they lack the ability to further increase their CBF in critical situations.^119^ Note that CVR can also be measured using other MRI techniques, for instance based on the blood-oxygen-level-dependent (BOLD) effect, which were reviewed elsewehere.^124^

Previous ASL studies in healthy aging and AD have critically been reviewed, whereby it was concluded that ASL is a promising tool to measure hypoperfusion in AD, contributing to early AD diagnosis.^125^ The hypoperfusion patterns found with ASL resembled fluorodeoxyglucose positron emission tomography (FDG-PET) derived hypometabolism patterns in AD and mild cognitive impairment.^126^ Furthermore, ASL demonstrated a decreased CBF in cSVD within WMHs,^127^ and perilesional zones,^128^ as well as in healthy aging.^129^ Nevertheless, CBF measurements by ASL should be interpreted with caution in these populations, since the measured hypoperfusion could have partially been caused by a longer ATT and thus might not entirely reflect functional rarefaction.^125^ One advanced ASL technique worth considering in cerebrovascular diseases is multi-delay ASL, which corrects the CBF for ATT at the cost of longer imaging duration or fewer label-control pairs (reducing the contrast effect) and more complex imaging processing.^122^ When multi-delay ASL is not feasible in populations with expected prolonged ATT, an ASL with long PLD is advised, including those where rarefaction is likely expressing.^130^

### Intravoxel incoherent motion imaging

**Figure 8.**
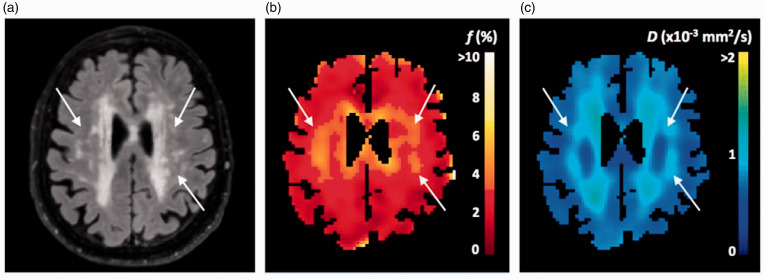
Example of IVIM parameter maps in cSVD: Fluid Attenuated Inversion Recovery (a), blood volume perfusion fraction (b) and parenchymal diffusion (c). The arrows indicate that even for normal-appearing white matter differences in f and D were visible. Source: reproduced with permission form publisher (2017).^134^ *f*: blood volume perfusion fraction; D: parenchymal diffusion; IVIM: intravoxel incoherent motion imaging; cSVD: cerebral small vessel disease.

Intravoxel incoherent motion (IVIM) imaging is a diffusion weighted imaging technique that can provide information about microvascular perfusion, in addition to the parenchymal diffusivity.^131^ When capillaries are assumed to follow a random pattern of directions, the flow of blood molecules through the capillary network can be referred to as pseudo-diffusion. The microvascular pseudo-diffusion is typically ten times higher than the parenchymal diffusivity.^132^ IVIM parameters can be obtained by fitting a biexponential model, resulting in the parenchymal diffusion ( D ), the microvascular pseudodiffusion ( D *), and the blood volume perfusion fraction ( f ). The product of the blood volume perfusion fraction and pseudodiffusion (fD*) is assumed to represent a proxy of the microvascular blood flow, though physical units differ.^133^ Examples of D - and f -maps for a patient with cSVD are visualized in Figure 8.^134^

The ability of IVIM to measure perfusion was validated by a study scanning healthy volunteers during normal breathing as well as hyperventilation.^135^ The IVIM perfusion measures ( f and fD*) were reduced in the hyperventilation condition, which was in agreement with hypotheses and with the measured ASL perfusion.^135^

Measuring acute hypoperfusion with IVIM was shown to be feasible in stroke patients, whereas the interpretation of the IVIM perfusion measurement in patient groups with chronic hypoperfusion is not straightforward.^136^ IVIM perfusion has previously been measured in cSVD,^136^ type 2 diabetes,^137^ AD ^138,139^ and neurologically normal aging subjects.^140^ These studies anticipated to find a reduced blood flow volume and/or hypoperfusion (lower f  and fD*), yet instead they found an increase in f  and fD* in the diseased/older group compared to the control group. These studies reasoned that the small blood vessels were dilated or that D * and f do not merely represent the microvascular component.^141^ Other factors may increase the attenuation of the microvascular component in IVIM and thus cause D * and  f to be higher, e.g. movement of interstitial fluid,^140,142^ BBB leakage,^137,143^ and vessel tortuosity.^144^ Since rarefaction is often linked with one or more of these vascular alterations, the IVIM perfusion measures (*f*, D* and fD*) are rather indicative of microvascular integrity than mere CBF.

## Other techniques to measure microvascular rarefaction

In this review we focused on MRI for the assessment of microvascular rarefaction since we believe it is the most feasible and versatile non-invasive imaging method to measure cerebral microvascular rarefaction in human in vivo. Other imaging modalities that can be used to measure CBF in human include positron emission tomography (PET), single photon-emission computed tomography (SPECT), and computed tomography (CT).^145^ PET can also be used to measure subtle BBB leakage.^146^ Although these other techniques can have specific advantages, they are less widely applied than MRI, as MRI provides detailed information on the brain’s anatomy and tissue lesions in the same examination session, can be more easily repeated over time, and involves no ionizing radiation exposure or administration of iodinated contrast material. Since microvascular rarefaction is assumed to be a systemic process, measures of rarefaction in other, more easily accessible, organs could serve as a surrogate marker for cerebral microvascular rarefaction. Especially measures of microvascular density in the retina, obtained by optical coherence tomography angiography, seem to be suited as surrogate marker due to the anatomical and functional similarity of the cerebral and retinal vessels.^147^ Other commonly used techniques to measure microvascular rarefaction include sublingual and skin intravital video-microscopy, and nailfold capillary microscopy. An extensive description of these non-MRI techniques is beyond the scope of this review.

## Discussion and future directions

Considering the growing ageing population, microvascular related brain disorders such as AD and VCI will become an even bigger worldwide health issue in the future then they already are today. Lack of early diagnosis and effective treatments are major problems. To enable early diagnosis and to find targets for new therapies, a better understanding of the underlying pathophysiological pathways leading to these microvascular brain disorders is needed.

In this review we have pointed out four main pathophysiological pathways involved in microvascular rarefaction (impaired angiogenesis and active capillary regression, endothelial dysfunction and apoptosis, pericyte loss, and loss of shear stress), and their possible interplay. Although the pathophysiological pathways underlying microvascular rarefaction in the brain are becoming clearer, there are still a number of knowledge gaps to be filled in. Currently, most research into the pathophysiology of microvascular rarefaction has been conducted in animal models, whereas studies in humans are needed to confirm that findings in animals relate to these in human. More research investigating the different pathophysiological processes simultaneously is needed to learn more about the sequence and the relative importance of these processes. Furthermore, little is known about the relation of microvascular rarefaction with other processes known to play a role in microvascular related brain disorders (e.g. neuro-inflammation, BBB disruption).

Existing MRI techniques can increase the knowledge about the role of microvascular rarefaction and its underlying pathophysiological pathways in microvascular brain disorders. The various techniques described in this review cannot depict microvessels in a morphological way, but can measure various functional aspects of microvascular rarefaction. Table 2 provides an overview of the main pathophysiological processes and the resulting expected changes in physiological MRI measures during the timelapse of rarefaction. Endothelial dysfunction causing a reduced arteriolar vasodilator capacity and enhanced pericyte contraction, both directly lead to a reversible reduction in blood flow and volume (i.e. functional rarefaction), whereas impaired angiogenesis and capillary regression, endothelial apoptosis and pericyte loss lead to an actual reduction in capillaries and thus an irreversible reduction in blood flow and volume. These reductions in blood flow and volume should be measurable with DSC MRI, ASL and IVIM. Vessel size imaging may be able to measure reduced mean vessel density. The CTTH determined with DSC MRI, can reflect the inhomogeneous flow patterns within the capillary network caused by pericyte loss. BBB leakage is not directly related to rarefaction itself, however the associated underlying pathophysiological processes influence the leakage measure *K^trans^* obtained with DCE-MRI. The interpretation of *K^trans^* (P · S) is not straightforward in the context of microvascular rarefaction. On one hand, a reduced capillary density leads to a reduced total vessel surface area S, contributing to a decreased leakage *K^trans^*. On the other hand, pericyte loss and endothelial apoptosis result in a leaky vessel wall, causing an increase in permeability P, resulting in an increased *K^trans^*. Previous studies have shown increased *K^trans^* in diseases associated with rarefaction,^114–116^ suggesting that the increase in P dominates. The detectable effects of vessel diameter changes in rarefaction by vessel size imaging are also debatable. The effects of endothelial dysfunction (i.e. reduced arteriolar vasodilator capacity) and enhanced pericyte contraction would lead to a decreased vessel radius. Meanwhile, there are other effects causing an enlarged mean vessel radius, e.g. vessel dilation as a compensatory mechanism for the reduced CBF in structural rarefaction or as a result of pericyte loss, and the occlusion or collapse of the smallest capillaries.^110^ Which techniques serve best to measure microvascular rarefaction at what stage of the pathological cascade in microvascular brain disorders is likely a complex puzzle to be solved.

In the context of microvascular brain disorders, the physiological MRI techniques described are mainly used in clinical research settings. These techniques can be classified into exogenous and endogenous contrast agent based techniques. In general, the strength of exogenous contrast agent based MRI techniques (i.e. DSC MRI, vessel size imaging and DCE MRI) is the strong contrast effect.^148^ However, contrast agents have a small risk to induce contrast nephropathy in patients with pre-existing renal impairment. The endogenous contrast agent based MRI techniques (i.e. ASL and IVIM) can be used as an alternative in people with contraindications for contrast agent, and are preferable when multiple measurements over time are required. The endogenous techniques are still emerging, and a detailed understanding of these techniques is needed for clear interpretation of the perfusion MRI measures in microvascular brain disorders. Researchers should be aware of the confounding factors, i.e. the influence of the arterial transit time on ASL perfusion signal and the non-perfusion factors that contribute to the IVIM perfusion signal.

Furthermore, the image acquisition and analysis protocols for advanced MRI techniques vary widely, which hampers progression in understanding the underlying pathophysiological pathways in microvascular rarefaction and its contribution to the clinical features of microvascular brain disorders such as AD and cSVD. To enable comparing studies over various sites, MRI protocols for image acquisition and image analysis need to be standardized.

More studies investigating these MRI techniques in human microvascular brain disorders are essential to establish test-retest repeatability and for validation of sensitivity to the subtle changes which can be found in these disorders. Ideally, longitudinal studies are needed to assess true rarefaction, i.e. temporal changes in microvascular density throughout disease progression and during healthy aging. Considering younger subjects are more likely to show significant differences in vessel density due to pathologies such as AD and cSVD in comparison with aged matched controls, it might be valuable to prevent oversampling of oldest-old subjects in future studies.^6^ Furthermore, there remains a need for validation of these MRI techniques in combined radiological-histological studies to confirm that the obtained MRI measures correlate with actual structural microvascular rarefaction. The international CRUCIAL consortium ^149^ aims to identify cerebral microvascular rarefaction using advanced MRI techniques in a population of VCI patients and healthy aged controls, and will validate these MRI techniques by scanning and performing histologic examination on rat brains.

We reviewed the evidence for microvascular rarefaction as an early pathophysiological process in a number of microvascular brain disorders and normal ageing, and the value of advanced MRI techniques addressing different properties of the microvasculature for its assessment. Although these MRI techniques are promising to contribute to early diagnostic information and to be used for monitoring treatment response in microvascular brain disorders in the future, these techniques are still emerging and do not provide disease specific information. More clinical studies, preferably longitudinal, are needed in large patient populations with various brain disorders as well as in aging.
